# Postoperative single-sequence (PoSSe) MRI: imaging work-up for CT-guided or endoscopic drainage indication of collections after hepatopancreaticobiliary surgery

**DOI:** 10.1007/s00261-021-02955-7

**Published:** 2021-02-15

**Authors:** Uli Fehrenbach, Timo A. Auer, Wenzel Schöning, Moritz Schmelzle, Christian Jürgensen, Thomas Malinka, Marcus Bahra, Dominik Geisel, Timm Denecke

**Affiliations:** 1 Department of Radiology, Charité – Universitätsmedizin Berlin, Klinik Für Radiologie, Augustenburger Platz 1, 13353 Berlin, Germany; 2 Department of Surgery, Charité – Universitätsmedizin Berlin, Campus Charité Mitte/Campus Virchow-Klinikum, Berlin, Germany; 3 Division of Hepatology and Gastroenterology, Medical Department, Charité – Universitätsmedizin Berlin, Berlin, Germany; 4 Department of Diagnostic and Interventional Radiology, Universitätsklinikum Leipzig, Leipzig, Germany

**Keywords:** Magnetic resonance imaging, Abbreviated protocol, Postoperative leakage, Hepatopancreaticobiliary surgery, Drainage

## Abstract

**Purpose:**

Fluid collections due to anastomotic leakage are a common complication after hepatopancreaticobiliary (HPB) surgery and are usually treated with drainage. We conducted a study to evaluate imaging work-up with a postoperative single-sequence (PoSSe) MRI for the detection of collections and indication of drainage.

**Material and methods:**

Forty-six patients who developed signs of leakage (fever, pain, laboratory findings) after HPB surgery were prospectively enrolled. Each patient was examined by abdominal sonography and our PoSSe MRI protocol (axial T2-weighted HASTE only). PoSSe MRI examination time (from entering to leaving the MR scanner room) was measured. Sonography and MRI were evaluated regarding the detection and localization of fluid collections. Each examination was classified for diagnostic sufficiency and an imaging-based recommendation if CT-guided or endoscopic drainage is reasonable or not was proposed. Imaging work-up was evaluated in terms of feasibility and the possibility of drainage indication.

**Results:**

Sonography, as first-line modality, detected 21 focal fluid collections and allowed to decide about the need for drainage in 41% of patients. The average time in the scanning room for PoSSe MRI was 9:23 min [7:50–13:32 min]. PoSSe MRI detected 46 focal collections and allowed therapeutic decisions in all patients. Drainage was suggested based on PoSSe MRI in 25 patients (54%) and subsequently indicated and performed in 21 patients (100% sensitivity and 84% specificity). No patient needed further imaging to optimize the treatment.

**Conclusions:**

The PoSSe MRI approach is feasible in the early and intermediate postoperative setting after HPB surgery and shows a higher detection rate than sonography. Imaging work-up regarding drainage of collections was successful in all patients and our proposed PoSSe MRI algorithm provides an alternative to the standard work-up.

## Introduction

One of the major complications of upper abdominal surgery (liver and pancreas) is the postoperative accumulation of fluid due to enteric, biliary, or pancreatic leakage [1, 2]. Such fluid collections can lead to hemorrhage or act as potential inflammatory foci and depending on their size, location, and the patient’s clinical status, they may have to be drained [3, 4]. Abdominal ultrasonography is the first imaging modality to detect postoperative fluid collections but is limited in identifying small but clinically important fluid collections especially after pancreatic surgery [5]. If sonographic evaluation is impaired but patients deteriorate clinically and inflammatory parameters continue to rise, computed tomography (CT), preferably contrast-enhanced (ceCT), is unavoidable for a reliable diagnosis [6]. If ceCT reveals significant postoperative fluid collections or abscesses, (percutaneous or transgastric) drainage insertion is indicated in the majority of patients [7]. In postoperative patients, the fast scan duration of CT is a major advantage over MRI. We here present a non-contrast, ultrafast abdominal, single-sequence MRI protocol, which we think is sufficient to detect postoperative fluid collections and their anatomic topography to decide on necessity and way of drainage. The decision whether a collection should be drained is made in collaboration with the clinician/surgeon and is based on the synopsis of symptoms (e.g., fever, pain), laboratory findings (e.g., leukocyte count, CRP level) and the accessibility, spread, and localization of the collection. The postoperative single-sequence (PoSSe) MRI approach we propose could replace a CT scan, thus dispensing with both radiation exposure and contrast medium administration. PoSSe MRI is characterized by a drastically reduced image acquisition duration (< 4 min), low specific absorption rate (SAR), and the patient’s overall time in the scanning room that might therefore be comparable to that of a contrast-enhanced CT examination.

The purpose of our study is to evaluate if a PoSSe MRI of the abdomen is feasible in the (early and intermediate [8]) postoperative setting and to evaluate its value in an imaging work-up for indications of CT-guided percutaneous drainage or transgastric drainage through endoscopy after hepatopancreaticobiliary (HPB) surgery.

## Material and methods

### Study design

The study was designed as a prospective cohort study. The study was approved by our hospital’s ethics committee (Internal reference: EA4/029/18), and written informed consent was obtained from all study patients.

Candidates for enrollment were fully oriented adult patients capable of giving a full written informed consent who recently underwent HPB surgery. Further inclusion criteria were elevation of inflammatory laboratory parameters (leukocyte count and/or level of c-reactive protein (CRP)) or clinical deterioration with an indication for imaging work-up. Exclusion criteria were contraindications to MRI, in particular non-MRI-compatible implants [9]. Study work-up of all patients included an abdominal ultrasound exam and PoSSe MRI of each patient. Therapy decision was primarily based on PoSSe MRI. If PoSSe MRI (+ sonography) was insufficient to plan drain insertion/further treatment, a ceCT was indicated according to the standard work-up.

### Abdominal sonography

All prospectively included patients underwent an abdominal ultrasonography for the detection of fluid collections, performed by a radiologist with at least 5 years of experience in sonography. The examiner was blinded to previous imaging findings and only knew the patient’s surgical record. In all patients, the whole abdomen was examined sonographically. Sonographic findings were reported according to the following study template. The abdomen was divided into 3 upper compartments: right (perihepatic), central (pancreatic), and left (perisplenic), and 1 lower compartment (pelvic). Each of the four compartments was evaluated for (1) insonation conditions (1: good, 2: impaired, 3: insufficient) and (2) the presence of focal or diffuse fluid collections. If a focal fluid collection was identified, the maximum diameter of the collection was measured. Each sonography examination was classified as to whether further imaging procedures were necessary for clinical decision making: 1: no evidence of a focal collection; 2: inconclusive sonography findings because of impaired conditions or uncertainty about fluid collection extent to be resolved by further imaging; and 3: unequivocal sonographic findings of focal fluid collections are accessible by drainage and no further imaging needed. Clinically relevant additional findings were recorded separately.

### PoSSe MRI

After abdominal ultrasound, every patient underwent an MRI scan at 1.5 T or 3 T scanners (MAGNETOM Aera/Skyra, Siemens Healthcare, Erlangen, Germany). The MRI examination was performed using phased-array body coils in all patients. The study protocol consists of localizer scans and an axial T2w half-Fourier acquisition single-shot turbo spin-echo (HASTE) sequence of the whole abdomen in a two-step acquisition approach (for patients taller than approximately 150 cm). The T2w HASTE sequence was acquired during breath-hold. HASTE images were acquired with following parameters (1.5 T/3 T): TR = 1400 ms/1600 ms; TE = 95 ms/95 ms; echo train length = 91/86; slice thickness = 6 mm/6 mm; matrix 320 × 260/320 × 250. The total examination duration from the patient entering the scanning room, preparation and placement of body coils, image acquisition, to leaving the scan room was measured. The acquisition time was recorded separately.

All MRI studies were analyzed in consensus by two radiologists with 5 and over 10 years of experience in abdominal MR imaging (*U.F.* and *T.D.*). The maximum diameters of all detected focal fluid collections (maximum of three collections) were measured, and the locations were classified as followed: 1. perigastric space, 2. pancreatic space, 3. perisplenic space, 4. perihepatic space, or 5. other. Each study was classified as to whether the imaging quality/information was sufficient for recommending clinical management (1: sufficient, no further imaging required; 2: insufficient, further imaging required; 2a: full protocol MRI, 2b: contrast-enhanced CT). If possible, an imaging-based management recommendation was made (1: Drainage possible; 1a: percutaneous access route, 1b: transgastric access route, 2: Fluid collection but no drainage possible or no fluid collection). A transgastric access route was proposed in cases with limited percutaneous access (e.g., overlying bowel structures). Clinically relevant additional findings were recorded.

### Evaluation of imaging work-up and indication of drainage

After imaging, indication of drainage was discussed with the physician/surgeon in charge. Clinical symptoms, laboratory parameters, and imaging results were considered in the decision-making process for indication. After indication, the best access route was discussed based on imaging. If the collection was accessible percutaneously, a CT-guided drainage was planned. If the imaging did not allow a safe percutaneous access route due to overlying bowel loops, the possibility of a transgastric drainage through endoscopy was evaluated with the gastroenterologist in charge. Imaging-based recommendations of PoSSe MRI (and sonography) were compared with the actually performed drainage procedure. If there was no indication for drainage, the patients’ clinical courses were monitored until discharge if any investigations besides conservative treatment were necessary. Based on the results, a PoSSe imaging work-up algorithm was evaluated.

### Statistics

Statistical analysis was performed using SPSS software version 25.0 (IBM; New York, USA). For the statistical results of the proportional distributions, contingency tables were used. Descriptive parameters are given as means and standard deviation. Accuracies are expressed as sensitivity and specificity levels.

## Results

### Patients

During the prospective study period of 1 year, 46 patients were included in the study. The majority of study patients (83%) had pancreatic surgery (*n* = 38) with 28 pancreaticoduodenectomies and 10 distal pancreatectomies combined with splenectomy. The other 8 patients (17%) underwent major hepatic surgery with 3 hemihepatectomies, 3 atypical resections and 2 right trisectionectomies with biliodigestive anastomoses (BDA). All patients presented with elevated inflammatory parameters (white blood cell counts and C-reactive protein). MRI examinations were 4 to 22 days after surgery, on average on the 10th postoperative day (POD). Patient characteristics are summarized in Table 1.

**Table 1:** Patient characteristics

	Number (%)/mean (SD)
Age (years)	58.46 (13.92)
Gender	
Female	21 (45%)
Male	25 (53%)
Surgery	
Pancreas	38 (83%)
Pancreaticoduodenectomy	28 (73%)
Distal pancreatectomy + splenectomy	10 (27%)
Liver	8 (17%)
Hemihepatectomy	3 (38%)
Trisectionectomy	2 (25%)
Atypical resection	3 (38%)
POD (days)	10.3 (4.2)
Laboratory	
White blood cells in 1/nl	14.09 (4.93)
C-reactive protein in mg/l	103.67 (68.71)

*POD* postoperative day

### Imaging findings

Sonographic conditions were classified as good in the perihepatic (96%), perisplenic (63%), and pelvic (94%) compartments. Sonographic evaluation of the central upper abdominal compartment was rated to be limited in 70% and insufficient in 13% of examinations. Sonography detected a total of 21 focal fluid collections. The average maximum diameter measured by sonography was 4.5 cm. Three patients had more than one focal collection detected in sonography. Diffuse fluid/ascites was present in 27 (59%) patients. Pleural effusion (*n* = 7) was the most common additional ultrasound finding. Cholestasis and a subcutaneous hematoma were detected as secondary findings in one patient each.

**Fig. 1:**
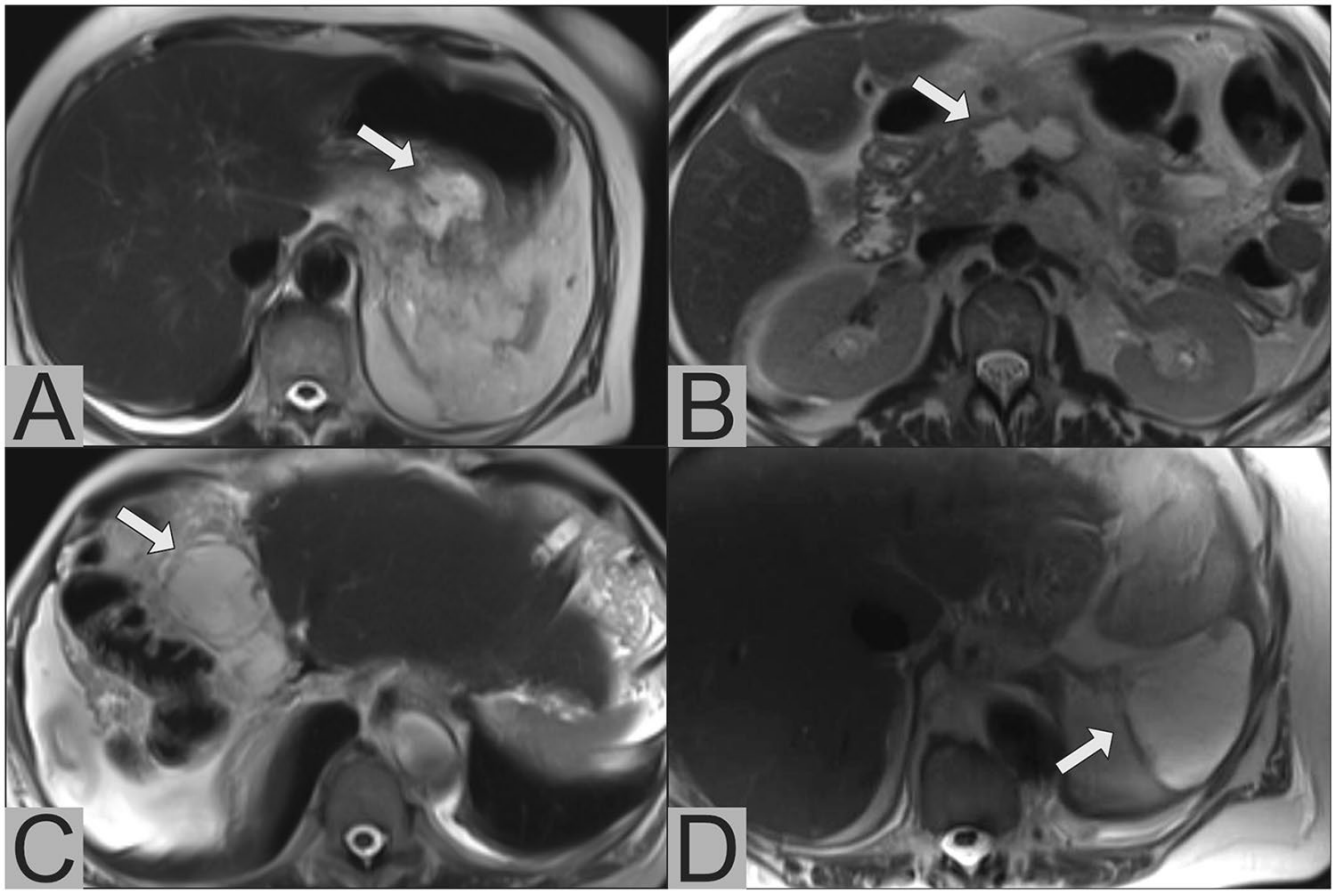
PoSSe MR images illustrating focal fluid collections (arrows) in different locations. **a** Collection in the perigastric space next to the anastomosis after pancreaticoduodenectomy and pancreaticogastrostomy; **b** collection in the pancreatic space after distal pancreatectomy; **c** collection in the perihepatic space after extended right hemihepatectomy; **d** collection in the perisplenic space after distal pancreatectomy with splenectomy

**Fig. 2:**
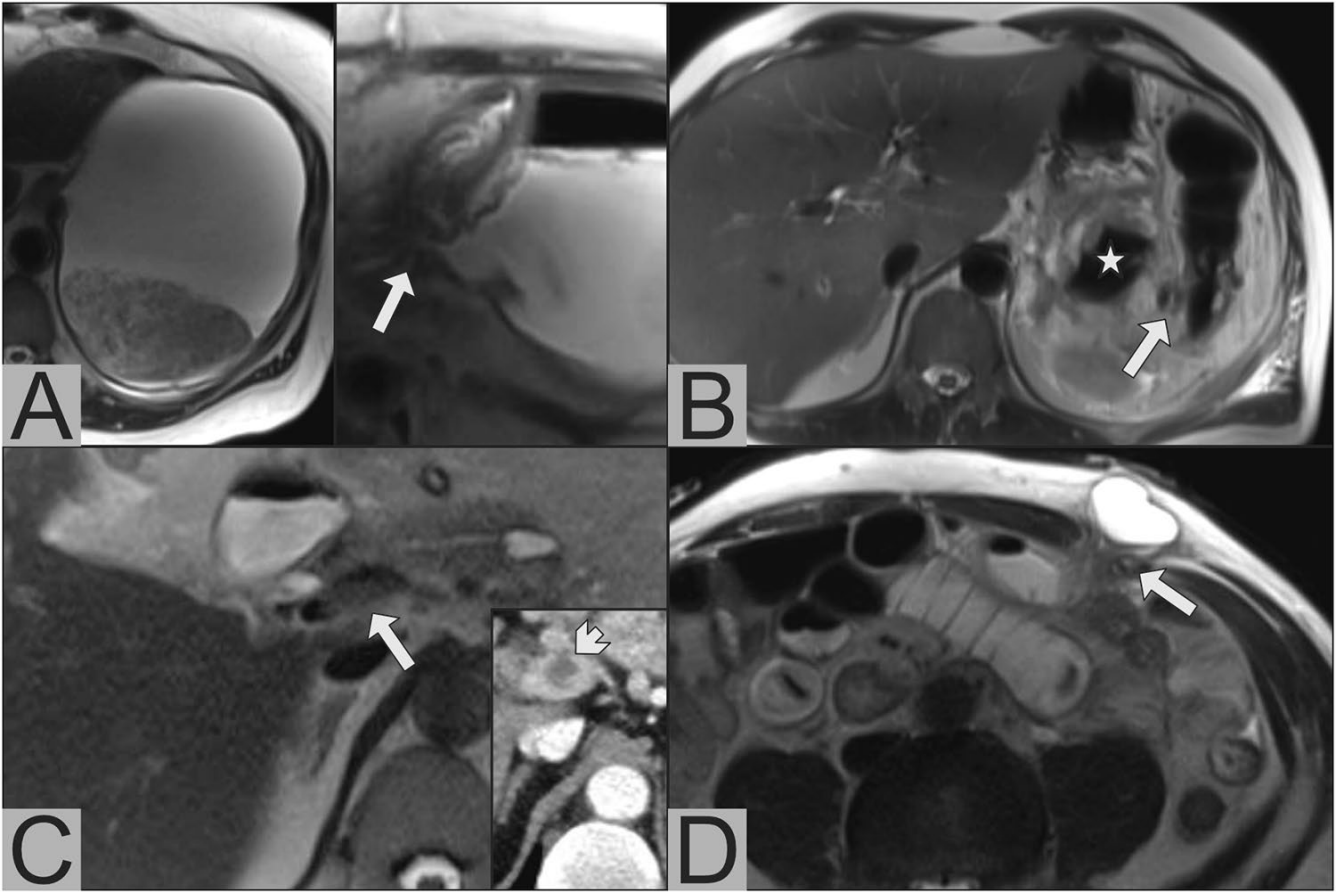
Important additional findings in PoSSe MRI; **a** Anastomotic stenosis of the gastrojejunostomy. Dilated and fluid-filled stomach on the left side. On the right side, narrow anastomosis (arrow) with prestenotic dilatation. **b** Bowel perforation. Perforated diverticula (arrow) of the left hemicolon with adjacent free gas in the peritoneal cavity (asterisk), these imaging findings were confirmed by subsequent surgery. **c** Partial portal vein thrombosis, characterized by missing flow void in PoSSe MRI (arrow), confirmation of thrombosis in ceCT (arrowhead). **d** Incarcerated hernia with a small hernial orifice (arrow)

All study patients completed the PoSSe MRI scan. The total acquisition time for PoSSe MRI was 3:30 min. The average time span from entering to leaving the scanning room was 9:23 min [7:50–13:32 min]. Image quality was rated sufficient in 100% of the cases. PoSSe MRI detected a total of 46 focal fluid collections. Nine patients had more than one focal fluid collection. The most common location was the perigastric space (*n* = 15) followed by the perihepatic (*n* = 14) and the pancreatic space (*n* = 12) (Fig. 1). Diffuse free fluid/ascites was found in 27 patients (59%). The average maximum diameter of the detected fluid collections measured by PoSSe MRI was 5.1 cm. The most common additional findings were pleural effusions (*n* = 17). Less common but important additional findings were: anastomotic stenosis (*n* = 3; Fig. 2a), bowel perforation (*n* = 1; Fig. 2b), partial portal vein thrombosis (*n* = 1; Fig. 2c), and incarcerated hernia (*n* = 1, Fig. 2d). Dislocation of intraoperatively inserted drains was detected in 2 patients. Sonographic and PoSSe MRI findings are summarized in Table 2.

**Table 2:** Imaging findings of focal collections in sonography and PoSSe MRI

Sonography	Perihepatic	Central	Perisplenic	Pelvic
Insonation conditions				
Good	44 (96%)	8 (17%)	29 (63%)	43 (94%)
Limited	2 (4%)	32 (70%)	17 (37%)	3 (6%)
Insufficient	0 (0%)	6 (13%)	0 (0%)	0 (0%)
Focal collections	11 (52%)	7 (33%)	3 (14%)	0 (0%)
Size in cm (SD)	4.98 (1.92)	4.11 (1.98)	3.70 (1.08)	–

PoSSe MRI	Perihepatic	Central		Perisplenic	Pelvic
		Pancreatic	Perigastric		
Focal collections	14 (30%)	12 (26%)	15 (33%)	5 (11%)	0 (0%)
Size in cm (SD)	5.39 (2.51)	5.13 (2.72)	4.71 (2.91)	4.60 (2.47)	–

For focal fluid collections detected by both modalities, sonography underestimated the size on average by 22% (2.0 cm) compared with PoSSe MRI.

### Evaluation of imaging work-up and indication of drainage

**Fig. 3:**
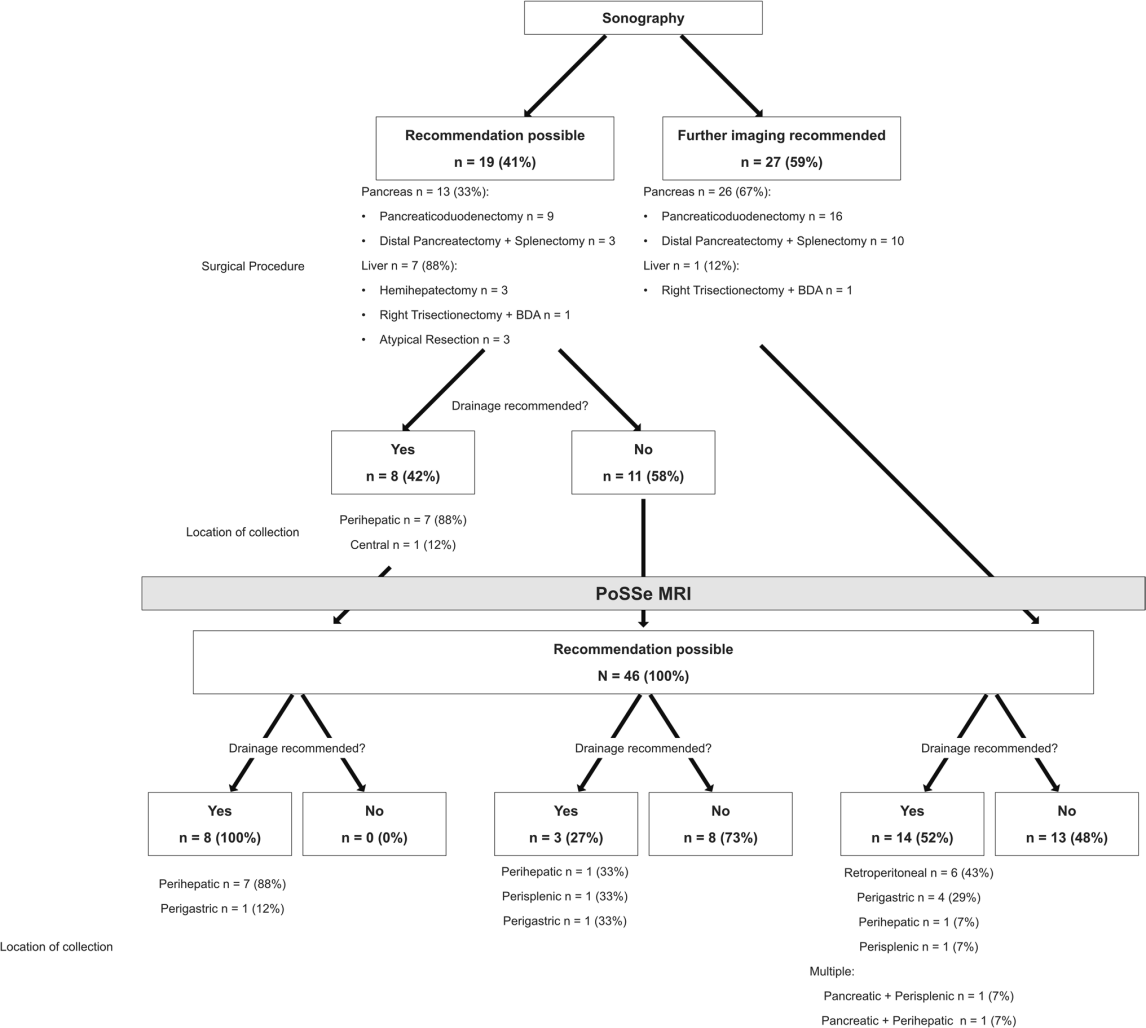
Evaluation of imaging work-up

The evaluation process is visualized in Fig. 3. In sonography as first-line modality, an adequate recommendation without further imaging was possible in 41% (*n* = 19) of the patients. Regarding the surgical procedure, sonography-based recommendations were possible in 33% (*n* = 13) of patients after pancreatic surgery and in 88% (*n* = 7) of patients after hepatic surgery. Sonographic findings led to a positive recommendation for drainage in 8 patients. Further imaging was recommended in 59% of patients (*n* = 27) because of poor overall sonographic conditions or uncertainty regarding the true extent of detected fluid collections.

PoSSe MRI studies allowed a recommendation regarding drainage to be made in all patients (100%). In patients with a sonography-based drainage recommendation (*n* = 8), the fluid collections were confirmed by PoSSe MRI in all cases. Regarding the patients with negative sonography findings (*n* = 11), PoSSe MRI detected drainable collections in 3 patients (27%). The sonographically missed drainable collections were located as follows: perihepatic in the liver hilum (*n* = 1), perisplenic (*n* = 1) and perigastric (*n* = 1). In patients, in which further imaging was recommended by sonography, PoSSe MRI proposed drain insertion in 14 patients (52%) with a total finding of 16 drainable collections. In the overall cohort, insertion of a drain was proposed in 25 patients. The other 21 patients showed no (drainable) collection neither in PoSSe MRI nor in sonography.

**Fig. 4:**
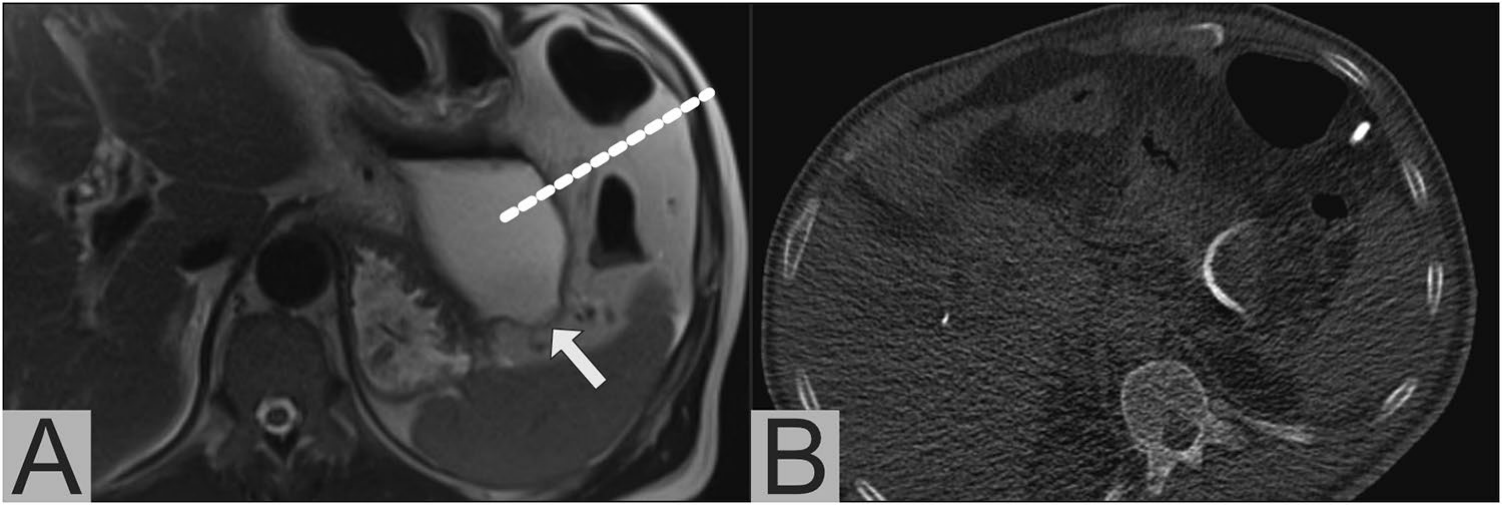
PoSSe MRI showing a perigastric collection (arrow) after pancreaticoduodenectomy, planning of CT-guided drainage insertion is marked by the dotted line (**a**); CT-Fluoroscopy showing the successful inserted drainage via the planned puncture tract (**b**)

Based on our analysis of imaging-based recommendations in relation to actually performed drain insertion procedures, we calculated 78% sensitivity and 90% specificity for sonography in the subset of patients in which a sonography-based recommendation was possible (41%). For PoSSe MRI, recommendation was possible in 100% of the patients and we calculated 100% sensitivity and 84% specificity. Complication-free drainage insertion was performed in 21 of 25 patients with a PoSSe MRI recommendation (17 of 20 CT-guided percutaneous, 4 of 5 transgastric endoscopic) (Fig. 4). In all 17 CT-guided drainage procedures, imaging findings of PoSSe MRI were confirmed by the planning non-contrast CT scan. All drained patients showed clinical improvement over time and no further intervention was necessary. Three of the other four patients with recommendation of drainage were surgically revised during their hospitalization. One patient was managed conservatively because of concomitant pneumonia and thrombocytopenia/impaired clotting function as contraindication for drainage procedures. All patients without drainage recommendation in PoSSe MRI recovered under conservative management until discharge.

## Discussion

**Fig. 5:**
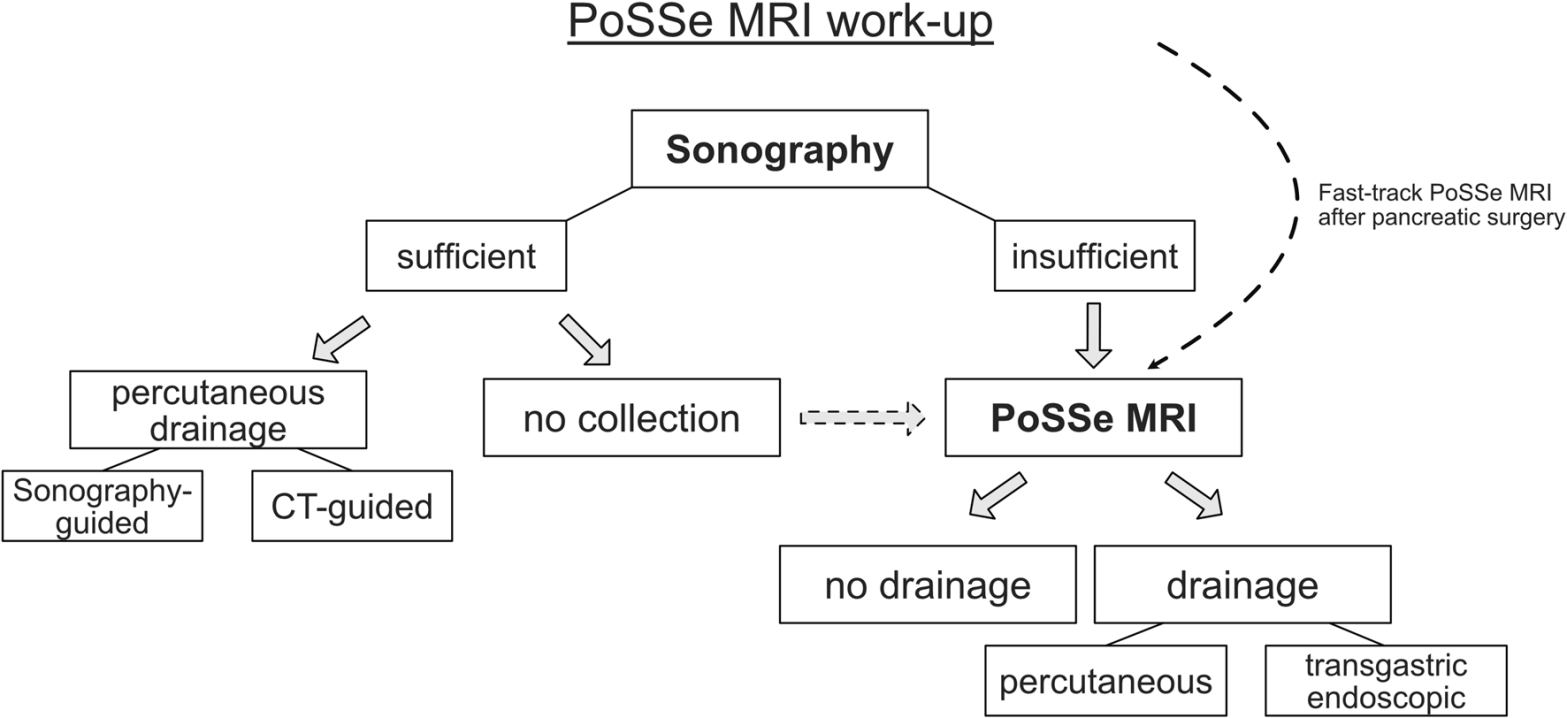
Proposal of PoSSe MRI work-up after HPB surgery

The ultrafast, single-sequence (T2w HASTE) MRI approach investigated here is feasible in postoperative patients after HPB surgery. The total time in the scanning room was around 10 min on average, which is comparable to the overall time of a ceCT study in our department. Our PoSSe MRI approach was shown to provide adequate images for the indication of drainage in all patients. No further imaging was required to decide if drainage was needed or not. PoSSe MRI imaging work-up allowed successful treatment of all study patients. Based on our results, we herein present a PoSSe MRI work-up algorithm for patients after HPB surgery (Fig. 5). In case of clinical suspicion of leakage, an initial sonography seems reasonable due to the wide availability and the possibility to perform the examination bedside. However, especially after pancreatic surgery, further cross-sectional imaging should not be delayed. Renouncement of sonography and fast-track PoSSe MRI should be considered after pancreatic surgery, due to the usually limited insonation conditions as shown in our evaluation. Our results suggest that patients with negative ultrasound findings, but at the same time with clinical suspicion of leakage, should undergo a PoSSe MRI without hesitation. In our cohort, this approach would have changed therapeutic management in 27% of patients with negative sonography findings. We propose that PoSSe MRI could be used as an alternative examination to CT in the further clarification of patients with insufficient sonography conditions.

In patients with delayed postoperative recovery or laboratory findings suggesting infection, surgeons suspect postoperative complications. Timely imaging and awareness of possible complications allow prompt initiation of appropriate management. Sonography may detect abnormal fluid collections in the surgical bed but is often impaired by overlying air in the stomach or transverse colon. These difficulties are more common in the early postoperative patient due to ileus, free air, and abdominal tenderness. That is why contrast-enhanced CT is often requested early and represents the “workhorse” imaging modality, enabling comprehensive assessment of the surgical site including detection of complications [10]. Abdominal MRI has established its value in the preoperative setting, allowing detailed tissue characterization in the upper abdomen (especially liver and also pancreatic). Postoperative MRI is not yet established as a routine modality although it has advantages that might be especially beneficial in postoperative patients. Patients after major abdominal surgery are at risk of developing postoperative acute kidney injury [11]. If contrast administration is to be avoided because of impaired renal function, the current alternative technique is non-contrast CT. Our data have shown that PoSSe MRI is suitable for postoperative management, thus providing another alternative when contrast administration is to be avoided. Safety concerns in the postoperative period have become negligible. Surgical clips are nowadays made of nonferromagnetic material. State-of-the-art fast MRI sequence acquisition techniques enable high image quality even in a patient with limited abilities to cooperate. Previous studies have shown that MRI has high sensitivity in the detection of abdominal fluid collections and that they indicate a surgical complication [12]. A recent study investigating postoperative complications in cholecystectomy recommends magnetic resonance cholangiopancreatography (MRCP) and also gadoxetic acid-enhanced MRCP to work-up suspected biliary complications and decide about the best management [10]. Especially in children, MRI should be preferred to avoid exposure to ionizing radiation. Lee et al. have demonstrated that a rapid non-contrast MRI scan is feasible and can identify drainable fluid collections in postappendectomy pediatric patients. Their protocol consisted of single-shot fast spin-echo, inversion recovery, and DWI sequences. [13] Our approach was to further reduce the acquisition time by restricting the protocol to a single T2w sequence. This ultrafast approach allows us to perform MRI examinations in patients who are in a poor condition immediately after HPB surgery. Besides the patients’ clinical condition, the decision when to drain is predominantly affected by the vicinity of a fluid collection to the anastomotic sites or major abdominal vessels. Moreover, it has been shown that contrast-related imaging features in ceCT (the standard imaging modality in the postoperative setting) are nonspecific and do not allow differentiation of non-infected and infected fluid collections in the majority of patients, so that a non-contrast examination as in PoSSe MRI seems sufficient [14].

Small perigastric fluid collections near pancreatic anastomoses can be difficult to drain percutaneously. Even small fluid accumulations in this location often require further investigation because of the increased risk of bleeding from adjacent arteries. If percutaneous drainage is precluded, these collections can be drained endoscopically in a transgastric approach [15]. Our study shows that PoSSe MRI enables reliable detection of these perigastric fluid collections and also the planning of a percutaneous or transgastric drainage strategy.

**Fig. 6:**
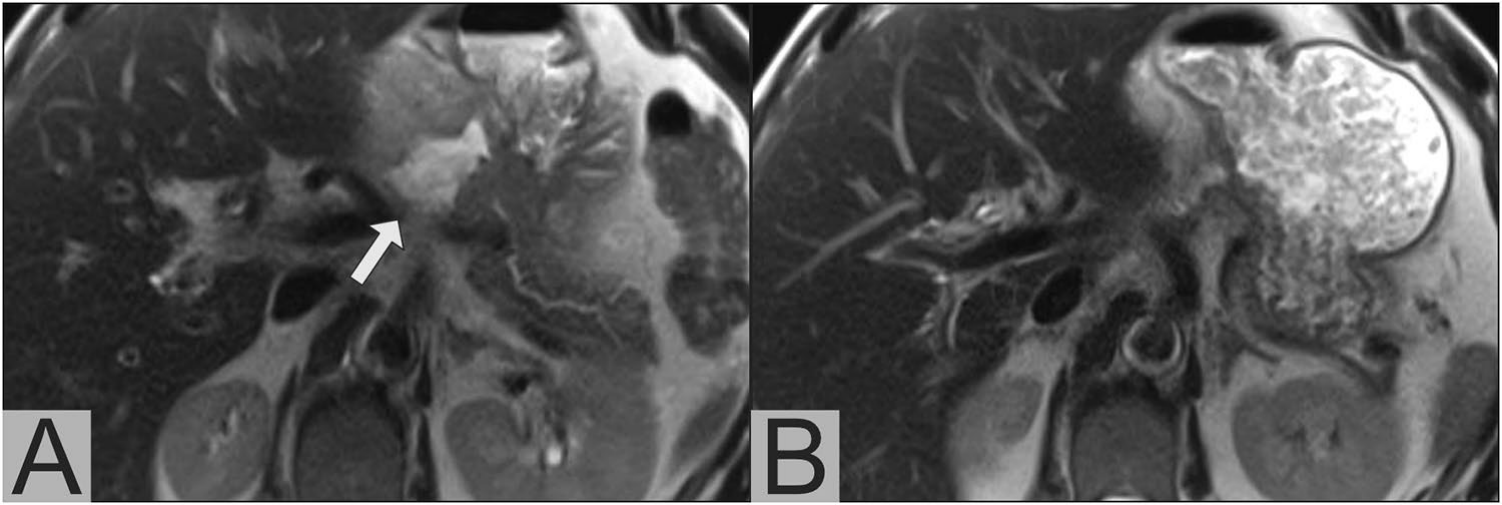
PoSSe MRI shows a small fluid collection next to the pancreaticogastrostomy (**a**, arrow). After consultation with the surgeon in charge no drain was placed in this collection. In the follow-up PoSSe MRI 5 days later (**b**), the collection has decreased in size and is barely visible

Fluid-sensitive T2w sequences have high sensitivity in the detection of fluid collections and edema. It has been shown that the majority of patients with an uneventful postoperative course develop edema in the gallbladder fossa after cholecystectomy [12]. Nevertheless, abdominal CT shows no abnormalities of the gallbladder fossa in about 80% of cases [16, 17]. Our results suggest that the situation is similar in patients after HPB surgery. Almost all patients in our study had mild edema or small fluid collections at anastomotic sites in PoSSe MRI. Even an experienced reader has to be aware of the increased sensitivity of these sequences and, if only little fluid is present, the decision regarding drainage should not rely on imaging alone. If imaging shows a small fluid collection and a patient has no clinical symptoms or laboratory evidence of infection, close follow-up imaging (e.g., repeat PoSSe MRI or focused ultrasound) should be preferred to drainage and a longer hospital stay (Fig. 6).

An observation we made in our study is that indwelling drains placed during surgery are more difficult to see by MRI than CT, and their identification depends on the reader’s experience. However, with knowledge of where drains have been placed by the surgeon, they are detectable in T2w MR images as well. In our study population, we accurately identified dislocated drains in two patients.

Another possible implication of PoSSe MRI, besides symptomatic postoperative patients, are high-risk patients without clinical suspicion of leakage especially in extended pancreatic surgery (e.g., Appleby procedure or robotic assisted pancreatic surgery) [18, 19]. Routine PoSSe MRI in these patients could prevent “failure to rescue” scenarios without contrast application or ionizing radiation as in ceCT [20, 21].

In general, costs of abdominal MRI are higher than those of abdominal CT examinations. Cost-effectiveness studies in abdominal imaging focus on the comparison of multiphase contrast-enhanced examinations in both modalities [22]. However, a recent cost-effectiveness analysis in pediatric patients with suspected appendicitis showed only minimal cost differences of ceCT and non-contrast MRI (full protocol) as initial imaging approach [23]. Zens et al. showed even lower costs of a “quick MRI” protocol compared to ceCT for the evaluation of intra-abdominal abscesses after acute appendicitis in pediatric patients [24]. The PoSSe MRI approach could make MRI even more cost-effective because of its drastically reduced acquisition time.

The major limitation of our study is the missing comparison with contrast-enhanced CT. During the study period, additional CT scans were not deemed necessary by the readers because of their diagnostic confidence based on PoSSe MRI and because of the already established high sensitivity of MRI in the detection of abdominal fluid collections [12]. In all cases, in which a CT-guided drainage procedure was performed, the planning CT scan confirmed the PoSSe MRI findings. The study was designed in such a way that only patients with inadequate PoSSe MRI work-up would receive a ceCT. Though, there was no need for, as all patients were adequately treated. The results of our study encourage to conduct a prospective randomized trial to address this missing comparison. Further limitations of our study are the small number of patients and their heterogeneity (pancreas and liver patients), which is attributable to the explorative nature of the study. The significance of postoperative fluid collections is different for these two patient groups. However, the ambition of the study was to show that PoSSe MRI is an alternative imaging modality after both liver and pancreatic surgery. Our initial results are encouraging and suggest that the PoSSe MRI protocol deserves further evaluation of its usefulness in larger, homogeneous patient cohorts. All readers were aware of the study design, which may have introduced detection bias. Our results cannot automatically be transferred to other radiological institutes. MRI examination slots are often more difficult to allocate than CT slots for capacity reasons, so that clinically urgent PoSSe MRI could be delayed. Therefore, the PoSSe MRI approach is also limited by the examination capacities of the individual institutes. In the case of postoperative patients, the condition and possibility to cooperate also limits the examination method (e.g., movement artifacts), so that critically ill, intensive care unit patients were not examined within the study, and the results are therefore not transferable to this patient group. All percutaneous drains in the study population were placed CT-guided due to the institute’s own preferences, so that no statement on the planning of sonography-guided drain placement is possible within the scope of the study. However, the study showed that PoSSe MRI is able to reliably identify sonographically limited visible collections so that a focused ultrasound examination or ultrasound-assisted drainage can be supported.

In conclusion, the PoSSe MRI approach is feasible in the early and intermediate postoperative setting after HPB surgery and has a higher detection rate than sonography. Imaging work-up regarding drainage was possible in all patients and our proposed imaging algorithm could provide an alternative to the standard work-up without the risks of radiation exposure or contrast-related adverse events.

## Abbreviations

ceCT: Contrast-enhanced computed tomography
HASTE: Half-Fourier acquisition single-shot turbo spin-echo
HPB: Hepatopancreaticobiliary
MRI: Magnetic resonance imaging
PoSSe: Postoperative single-sequence
T2w: T2-weighted
TE: Echo time
TR: Repetition time
